# Pregabalin-induced urticarial rash and neutropenia in a renal transplant recipient: a case report

**DOI:** 10.1186/s12882-019-1401-3

**Published:** 2019-06-06

**Authors:** Sanjeev Sahota, Robin Geoffrey Parry, Giorgio Gentile

**Affiliations:** 1 Department of Nephrology, Royal Cornwall Hospitals NHS Trust, Truro, Cornwall UK; 2 University of Exeter Medical School, Exeter, UK; 3 Department of Internal Medicine, University of Perugia, Perugia, Italy

**Keywords:** Pregabalin, Neutropenia, Rash, Drug reaction, Renal transplant

## Abstract

**Background:**

Pregabalin is a medication used to treat epilepsy, neuropathic pain and generalised anxiety disorder. The most common side effects of pregabalin include dizziness, drowsiness, weight gain, ataxia and diplopia. On the other hand, neutropenia and rash are rare side effects of pregabalin, and at the time of writing, there are only two documented cases of neutropenia and one of rash in the literature, none of which involved renal transplant recipients.

**Case presentation:**

We present a 37-year-old renal transplant recipient who was admitted with lethargy, sore throat, urticarial rash and neutropenia after recently being commenced on pregabalin. On physical examination, he had erythematous urticarial rash near his renal transplant scar, on his right elbow, left knee and left wrist. Bacterial/viral serology and immunology were all negative. A blood film confirmed neutropenia and revealed reactive lymphocytes and neutrophil left shift, and those features were compatible with drug reaction. After cessation of the pregabalin, the neutropenia resolved. No other causes of neutropenia or urticarial rash were identified.

**Conclusion:**

To the best of our knowledge, we have described the first case of concomitant pregabalin-induced neutropenia and urticarial rash in a kidney transplant patient. This case report highlights the importance of close monitoring when starting any new medications, particularly in the immunosuppressed population, and is relevant because of the growing usage of pregabalin for treating neuropathic pain in such patients and the risk that a missed pregabalin-related neutropenia could lead to unnecessary modifications of the immunosuppressive treatment.

## Background

Pregabalin is a widely used antiepileptic medication which is also licensed for the treatment of neuropathic pain [1]. Chemically it is an analogue of the inhibitory neurotransmitter, gamma-aminobutyric acid (GABA) [2–5]. Its mechanism of action is mediated by α2δ subunit of presynaptic P/Q-type Calcium channels, which reduces axonal aspartic and glutamic acid leading to reduced α-amino-3-hydroxy-5-methyl-4-isoxazolepropionic acid receptor (AMPA) activation on noradrenergic nerve terminals [1, 6, 7]. The most common side effects of pregabalin include dizziness, drowsiness, diplopia, weight gain and ataxia. Neutropenia and urticaria have been cited as uncommon side effects, each occurring in 1/100–1/1000 of patients [8].

Pregabalin is a pro-drug to gabapentin and both drugs have got similar effectiveness in diabetic peripheral neuropathy, post herpetic neuralgia and partial onset seizures. However, while gabapentin is a recognised cause of neutropenia [3, 9, 10], to the best of our knowledge there are only two reported cases of pregabalin-induced neutropenia, [11, 12] and none in patients with renal disease.

We report the first case of a renal transplant recipient who developed concomitant neutropenia and urticarial rash after starting pregabalin for bilateral leg pain.

## Case presentation

A 37-year-old Caucasian renal transplant recipient was admitted with a two-day history of lethargy, sore throat, abdominal tenderness around his transplant site, and arthralgia in his elbows, knees and ankles. Regarding his past medical history, he had focal segmental glomerulosclerosis which eventually led to end-stage renal disease for which he received a first renal transplant in 2000. This gradually failed due to chronic allograft nephropathy, and he then underwent a further transplant in 2011, which required an angioplasty in 2013 due to transplant renal artery stenosis. He also suffered from asthma. Prior to admission, there was no history of recent foreign travel, or contacts with anybody with recent foreign travel or similar symptoms. His medication list included tacrolimus, azathioprine, prednisolone, aspirin, omeprazole and calcium carbonate plus cholecalciferol. He had been started on pregabalin by his general practitioner (GP) 2 weeks prior to admission because of a two-month history of severe pain in his shinbones, which was interpreted as neuropathic pain. He had no known drug allergies.

On physical examination, the patient had some discrete patches of urticarial rash, including a 12 × 8 cm erythematous urticarial rash overlying his renal transplant scar, which was tender on palpation, and similar smaller circular areas of erythema on the back of his right elbow, left knee and left anterior wrist. Chest was clear, heart sounds were normal with no murmurs, blood pressure and heart rate were within normal limits, and he was afebrile.

Given the clinical presentation and the patient’s past medical history, the initial differential diagnosis was broad and included malignancy, autoimmune causes of erythema nodosum, and infections, including Lyme disease, syphilis and other atypical infections, which are more prevalent in the immunosuppressed patient. Diagnostic work-up included mid-stream urine dipstick, chest X-ray, full blood count, liver and renal function tests, electrolytes, cytomegalovirus (CMV), Epstein-Barr virus (EBV), polyomavirus BK, syphilis screen, anti-hepatitis B and C antibodies and Borrelia serology.

Preliminary results showed a white cell count of 2.4, with neutrophils at 1.08 (45%) [Table 1]. He was therefore started on amoxicillin and gentamicin after discussion with the microbiology team, given the chronic immunosuppressive treatment, the presence of lethargy and a moderately raised CRP. Antibiotic treatment was discontinued after 2 days following a negative blood culture.

**Table 1:** White cell count before pregabalin initiation, at day 1 post-admission (pregabalin was discontinued on admission), and up to day 9 post-admission

	Before pregabalin	Day 1	Day 3	Day 4	Day 6	Day 9	*Reference Range*
White Cell Count (10*9/L)	7.4	2.4	2.9	3.1	3.2	4.6	*3.9–11.1*
Neutrophils (10*9/L)	6.22 (84%)	1.08 (45%)	0.20 (6%)	0.27 (8%)	0.56 (17%)	2.96 (64%)	*1.7–7.5*
Lymphocytes (10*9/L)	0.9 (12%)	0.91 (38%)	2.10 (72%)	2.18 (70%)	2.04 (63%)	1.34 (29%)	*1–3.2*
Monocytes (10*9/L)	0.21 (2%)	0.29 (12%)	0.36 (12%)	0.38 (12%)	0.34 (10%)	0.19 (4%)	*0.2–0.6*
Eosinophils (10*9/L)	0.05 (0%)	0.1 (4%)	0.22 (7%)	0.24 (7%)	0.22 (7%)	0.09 (2%)	*0.03–0.46*
Basophils (10*9/L)	0.01 0%	0.02 (0%)	0.02 (0%)	0.02 (0%)	0.03 (0%)	0.02 (0%)	*0.02–0.1*
Haemoglobin (10*9/L)	148	141	131	148	141	145	*120–160*
MCV (fL)	103.9	98.3	105.6	103.2	103	101.4	*77–98*
MCH (pg)	35.9	35.6	34.5	34.6	34.8	34.8	*27.3–32.6*
Platelets (10*9/L)	227	237	214	250	223	235	*150–400*
CRP (mg/L)	6.7	16	12	6.5	5	2.4	*0–5*

The pregabalin was stopped on admission, and the neutrophil count continued to drop over the next day, but slowly began to recover over the subsequent few days. Azathioprine treatment was continued at the same dosage throughout the admission.

**Fig. 1:**
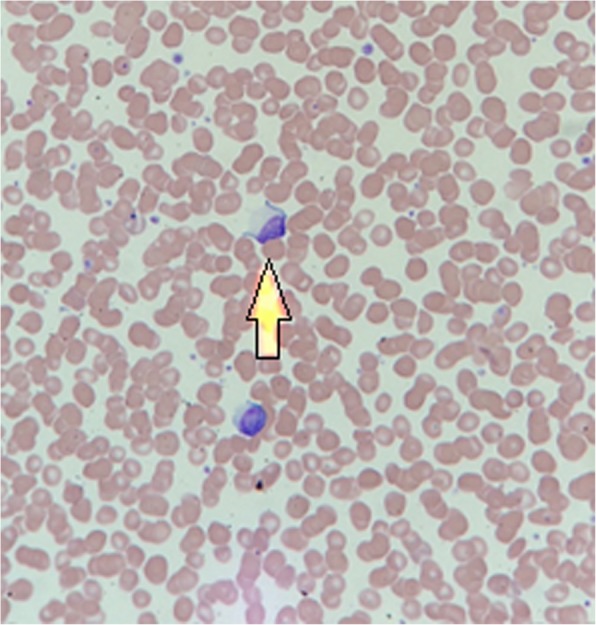
arrow pointing to a reactive lymphocyte, an immature lymphocyte with a larger nucleus with basophilic cytoplasm and “scalloped” cell membranes

**Fig. 2:**
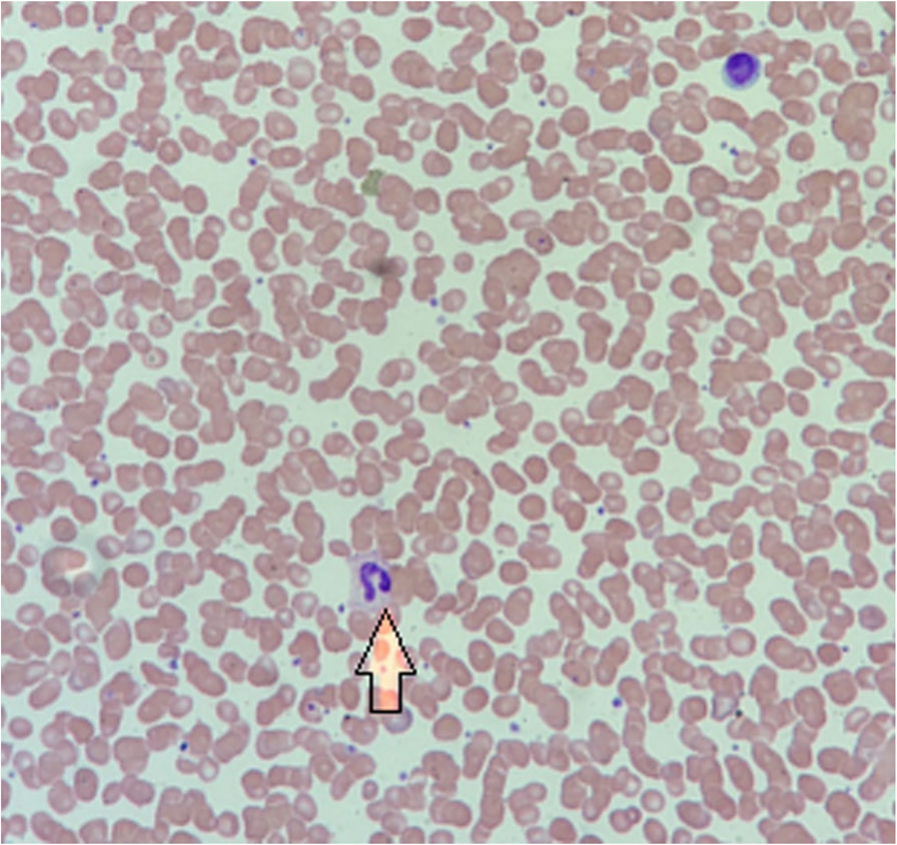
arrow pointing to a left-shifted neutrophil. This is denoted by fewer lobes separated by a fine filament bridge, and represents a response where immature neutrophils are released into the peripheral circulation

A subsequent blood film (Figs. 1 and 2) confirmed severe neutropenia and revealed some reactive lymphocytes and neutrophil left shift, which was interpreted by the haematologists as likely drug reaction. Immunological results showed normal C3 (1.07, reference range 0.75–1.65) and C4 (0.21, reference range 0.14–0.54), and normal IgG, IgM, and IgA. CMV and BK viral loads, and serology for syphilis, Borrelia, Hepatitis B and C, and HIV were all negative. EBV serology was negative. Cytoplasmic speckling was seen on Hep-2 cells, but the extractable nuclear antigen screen (ENA) antibodies that followed were negative. An ultrasound of the transplanted kidney was normal. Renal function remained stable during the hospital stay. The patient continued to be monitored on the ward and after 9 days as an inpatient, his white cell count became normal.

## Discussion and conclusions

Renal transplant patients are at risk of neurological complications, occurring in around 6–21% of patients, with infections being the most common cause [13–15]. Among the different neurological conditions, neuropathic pain has a prevalence of 1–2% [16–19].

The differential diagnosis of our patient when he first presented was wide, and included haematological malignancies, infectious diseases including Lyme disease, Epstein Barr virus, Cytomegalovirus, syphilis, autoimmune disorders and a drug reaction secondary to azathioprine. Sepsis was an important initial differential diagnosis due to the patient’s immunosuppression, however a lack of fever, normal physiological observations, a mildly raised C-reactive protein and negative cultures reduced the probability of this diagnosis.

Azathioprine has been known to cause arthralgia, bone marrow suppression, leukopenia, myalgia and rash [20]. It is important to note that the azathioprine dose remained unchanged for years, hence azathioprine toxicity was not included in the differential diagnosis.

As all other causes of the patient’s signs and symptoms were excluded, and both the neutropenia and the rash completely resolved after cessation of the pregabalin, this was deemed the causative factor of the clinical presentation. The blood film showed reactive lymphocytes, which can be due to a wide range of causes such as viral illnesses, endocrine pathologies such as Addison’s disease, and drug reactions. The blood film also showed a left shift of neutrophils, which indicates immature neutrophils. Although the marrow picture in drug-induced neutropenia is variable, typically immature neutrophils are relatively abundant compared to mature marrow neutrophils, which is partly explained by an expansion of the immature myeloid compartment due to the higher demand for more circulating neutrophils. The above findings in conjunction with negative microbiology helped to support the diagnosis of pregabalin-induced drug reaction. The patient has since remained off pregabalin, and his follow up blood tests show all blood cells within the normal range. Due to his severe idiosyncratic reaction, we have been reluctant to begin any new medications, and currently his neuropathic symptoms are managed conservatively.

In our case report, pregabalin was discontinued straight away because of the short time frame from initiation (2 weeks) and the very low risk of withdrawal symptoms. In patients on long-term treatment, pregabalin should be gradually tapered down over a period of 1 week.

At the time of writing, there are 2 cases of pregabalin associated neutropenia. Comparing the previously described cases, the presentation of pregabalin-induced neutropenia appears to be idiosyncratic, with one being a 64-year-old diabetic hypertensive presenting with fever and malaise with no rash, [12] and the other, a 45-year-old fit and well lady presenting with acute onset aggressive behavior [11]. In the former, the neutrophils dropped after 3 weeks of pregabalin use, returning to normal after 1 week, and the latter showing a drop in neutrophils after 2 days, returning to normal after 10 days. Both previous cases demonstrated a similar timeframe of recovery for the white blood cell and absolute neutrophil count to return to normal as our described case. Neither previous case describes a rash, however one case describes general lethargy and malaise, similarly to our patient. There is also one case of rash associated with pregabalin use [21]. However, this case describes a widespread erythematous maculopapular rash which required antihistamines and steroids for resolution, whereas our patient had discrete patches of urticarial rash which resolved on cessation of pregabalin without the use of further immunosuppressive agents or antihistamines.

To the best of our knowledge we have described the first case of concomitant pregabalin-induced neutropenia and urticarial rash in kidney transplant recipients. Pregabalin-induced neutropenia is uncommon, and failure to recognize it might lead to unnecessary changes in the immunosuppressive regime or in prophylactic drugs like co-trimoxazole or valganciclovir. This case highlights the importance of monitoring routine blood tests, including the full blood count, when starting new medications, especially in those who are immunosuppressed.

## Data Availability

Not applicable.

## Abbreviations

AMPA: α-amino-3-hydroxy-5-methyl-4-isoxazolepropionic acid receptor
CRP: C-reactive protein
eGFR: estimated glomerular filtration rate
GABA: Gamma-aminobutyric acid
GP: General practitioner
MCH: Mean corpuscular haemoglobin
MCV: Mean corpuscular volume
